# The effect of increased frequency of hemodialysis on vitamin C concentrations: an ancillary study of the randomized Frequent Hemodialysis Network (FHN) daily trial

**DOI:** 10.1186/s12882-019-1311-4

**Published:** 2019-05-17

**Authors:** Jochen G. Raimann, Samer R. Abbas, Li Liu, Brett Larive, Gerald Beck, Peter Kotanko, Nathan W. Levin, Garry Handelman, the FHN Trial

**Affiliations:** 1 Renal Research Institute, 315 East 62nd Street, 4th Floor, New York, NY 10065 USA; 2 Renal Division, Peking University First Hospital, Beijing, People’s Republic of China; 3 Key Laboratory of Renal Disease, Ministry of Health of China, Beijing, China; 4 Key Laboratory of Chronic Kidney Disease Prevention and Treatment, Ministry of Education, Beijing, China; 5 Cleveland Clinic Foundation, Cleveland, OH USA; 6 Icahn School of Medicine at Mount Sinai Health System, New York, NY USA; 7 University of Massachusetts Lowell, Lowell, MA USA

**Keywords:** Frequent hemodialysis network (FHN), Hemodialysis, Clinical trials, Left ventricular mass, Physical health component score, More frequent hemodialysis, Vitamin C

## Abstract

**Background:**

Reports on vitamin C in HD patients have shown effects of vitamin C deficiency in association with scurvy symptoms. Dialyzability of water soluble vitamins is high, and substantial losses in those who are dialyzed more frequently were hypothesized. The randomized FHN Daily Trial compared the effects of in-center HD six versus three times per week. We studied baseline correlations between vitamin C and potentially associated parameters, and the effect of more frequent HD on circulating vitamin C concentrations.

**Methods:**

We studied vitamin C levels at baseline and months, 3, 5 and 11. Patients enrolled between 2007 and 2009 into the randomized FHN Daily trial in the East Coast consortium were approached for participation. Predialysis plasma samples were processed with metaphosphoric acid and frozen at − 70 °C for measurement with HPLC. Regression models between baseline log-transformed vitamin C and hemoglobin, CRP, eKt/V, ePCR and PTH, and a linear mixed-effects model to estimate the effect size of more frequent HD on plasma vitamin C, were constructed.

**Results:**

We studied 44 subjects enrolled in the FHN Daily trial (50 ± 12 years, 36% female, 29% Hispanics and 64% blacks, 60% anuric). Vitamin C correlated significantly with predialysis hemoglobin (r = 0.3; *P* = 0.03) and PTH (r = − 0.3, *P* = 0.04), respectively. Vitamin C did not significantly differ at baseline (6×/week, 25.8 ± 25.9 versus 3×/week, 32.6 ± 39.4 μmol/L) and no significant treatment effect on plasma vitamin C concentrations was found [− 26.2 (95%CI -57.5 to 5.1) μmol/L at Month 4 and − 2.5 (95%CI -15.6 to 10.6) μmol/L at Month 12.

**Conclusions:**

Based on data from this large randomized-controlled trial no significant effect of the intervention on circulating plasma vitamin C concentrations was found, allaying the concerns that more frequent HD would affect the concentrations of water-soluble vitamins and adversely affect patient’s well-being. Correlations between vitamin C and hemoglobin and PTH support the importance of vitamin C for normal bone and mineral metabolism, and anemia management.

## Background

Low plasma levels of vitamin C have been documented in some hemodialysis patients for many years, and levels sometimes decrease to the range associated with scurvy (ca 1–2 μmol/L) [1]. This has led to the recommendation for use of moderate doses of supplements, which are prescribed in some patients [2].

What is the cause of these low levels? The most common vitamin C-rich foods, citrus and strawberries, also contain abundant potassium or oxalate [3], and are strictly limited in diets prescribed by renal dietitians [4]. Compliant patients have very low intake of such foods, which provide much of the vitamin C in the American diet. Although supplements are often prescribed, the dose of the supplement is usually limited to 60–100 mg/day, because of concern of conversion of excess vitamin C to oxalate (and subsequent calcium-oxalate deposition in soft tissue), and clinicians are conservative with use of supplemental vitamin C. Inflammation may be a factor causing low vitamin C levels and deficiency has been observed in patients with normal renal function during acute illness [5–8].

The dialysis process itself must be considered; during hemodialysis, vitamin C is readily cleared, because it is a soluble, low-MW substance, and amounts of 50 mg or greater can be removed by a single treatment [9]. Plasma vitamin C at the end of a single treatment is typically decreased by two-thirds, in parallel with urea and creatinine [10]. The high dialytic clearance of vitamin C has led to the hypothesis that the treatment itself could be the primary cause of low vitamin C levels.

More frequent dialysis could achieve even greater removal of vitamin C, and lead to a more severe deficient state. This is of concern because low vitamin C is linked with difficulties in management of renal anemia [1] and may also be associated with other negative outcomes, possibly increasing the increased risk of mortality [11, 12].

Although there have been several trials of more frequent or prolonged hemodialysis [13, 14], there has been no examination of the effects of this modality on plasma vitamin C levels. We report here on plasma vitamin C levels and its changes in a group of patients treated 6×/week with short daily HD in the Frequent Hemodialysis Network Daily Trial, compared with controls. As a secondary outcome, we examined vitamin C in relation to hemoglobin levels, C-reactive protein (CRP), and parathyroid hormone levels (PTH).

## Methods

### Study design, setting and participants

The FHN Daily Trial (http://www.clinicaltrials.gov #NCT00264758) enrolled from 01/2006 to 03/2010, and patients enrolled during 07/2006 to 03/2010 at nine of the participating centers (listed in Appendix/Recruitment sites) were approached for participation in this ancillary study to the main trial. A detailed overview of the trial design, the inclusion and exclusion criteria and randomization of the main trial has been published [15]. The study was approved by the Institutional Review Board of Beth Israel Medical Center and was conducted according to the Declaration of Helsinki. All patients signed informed consent.

This ancillary study aimed to recruit all patients that were enrolled as subjects during the entire active study period into the prospective, randomized, controlled FHN Daily Trial. Patients enrolled in the main trial were randomized on a facility level to receive either more frequent (6 times per week) or a conventional (3 times per week) HD regimen. The two co-primary outcomes of the main trial were the composite between mortality and left ventricular mass reduction and change in Physical Health Component score as per Short Form-36, respectively. All subjects recruited in the East Coast consortium were approached for recruitment for our ancillary study studying vitamin C concentrations throughout the course of the study. Due to the randomization on a facility basis in the main trial and our approaching of all patients enrolled in the main trial, the study population of this ancillary study can be considered as being virtually randomized to treatment arm allocation.

### Measurements

Baseline demographic parameters were obtained in the FHN Daily Trial at baseline. Information on vitamin-supplements were assessed at baseline and monthly into the study. Hemoglobin, CRP, blood urea nitrogen and PTH were collected as per protocol of the main trial. Equilibrated non-normalized protein catabolic rate, equilibrated per session and weekly standard Kt/V were computed as per the FHN Daily Trials protocol. Physical Health Composite was assessed according to the Short Form-36 administered following the protocol of the main trial. Vitamin C samples were collected prior to a mid-week treatment coinciding at baseline, months 3, 5 and 11. After collection, plasma samples were processed with metaphosphoric acid and frozen at − 70 °C for measurement with high-performance liquid chromatography [16] at the study laboratory (University of Massachusetts, Lowell, USA).

### Statistical analyses

Vitamin C as the parameter of primary interest was analyzed as a continuous variable (natural log-transformed due to skewness) and dichotomized as deficient/non-deficient as per a threshold of 10 μmol/L [17]. All reported *p-*values are 2-sided without adjustments for multiple comparisons. *P*-values were considered significant when below 5%. We conducted all analyses using SAS version 9.2 (SAS Institute, Cary, NC).

### Comparative analysis

For the comparison between changes in parameters over time and the quantification of the treatment effect of the intervention we employed linear mixed-effects models adjusted for the baseline values of the studied parameter, which allowed us to estimate the effect size of more frequent HD on plasma vitamin C. Due to the limited sample size we refrained from additional adjustments of the models as done in the main trial.

### Correlational analysis

Pearson and Spearman regression models between baseline log-transformed vitamin C and hemoglobin, CRP, eKt/V, ePCR and PTH, were constructed for both the 3 times and 6 times per week group, and for the overall cohort.

## Results

**Fig. 1:**
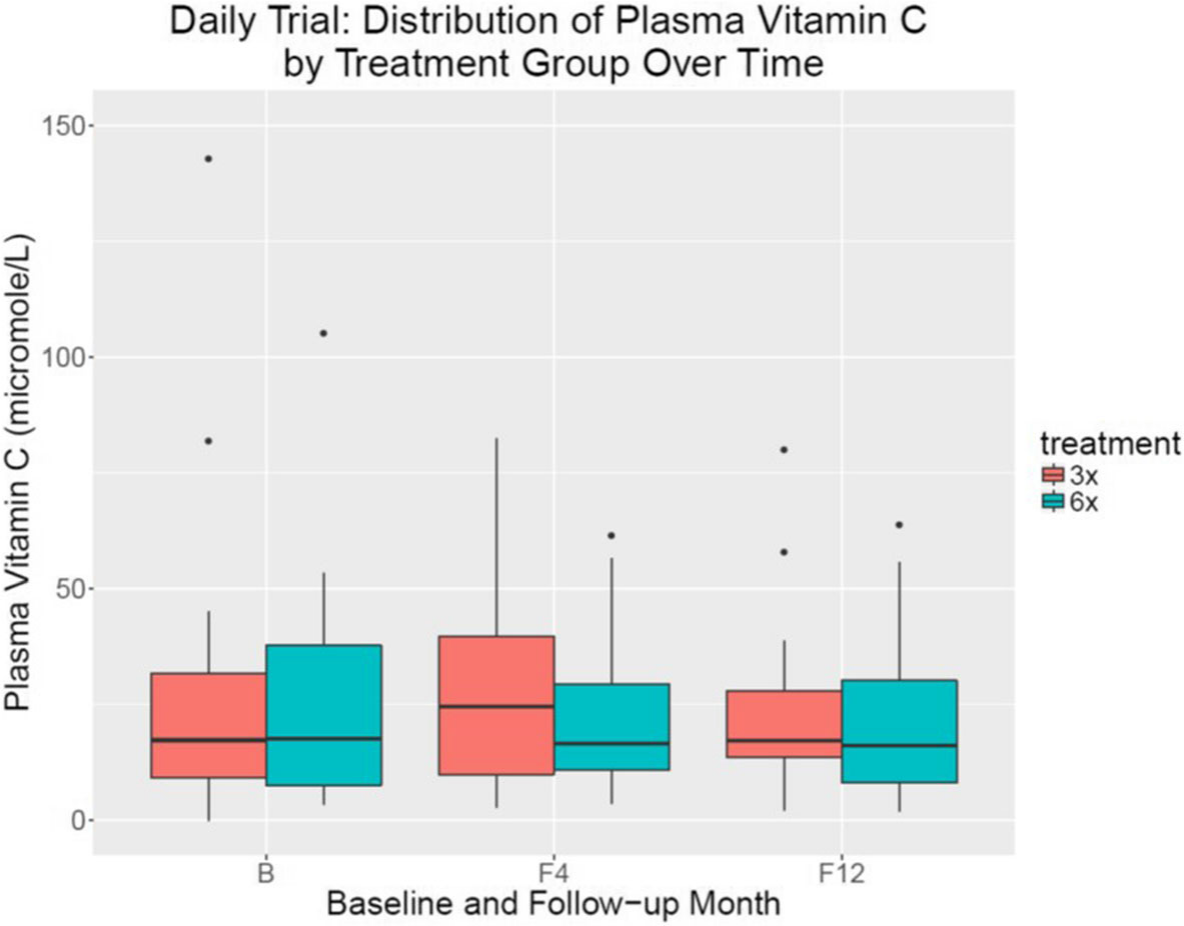
Box-Whisker plot depicting the distribution of plasma vitamin C by treatment group over time in the study population

We studied 44 subjects enrolled in the FHN Daily trial (50 ± 12 years, 36% female, 29% Hispanics and 64% blacks, 60% anuric; see Table 1 for detailed demographics) from 07/2006 to 03/2010 and, of those, we were able to follow 30 until the end of the study (13 controls and 17 in the intervention group). There were no relevant differences in vitamin C supplementation in both groups at baseline and during the course of the study (Table 2). Vitamin C concentrations did not significantly differ at baseline (6×/week: 25.8 ± 25.9 μmol/L versus 3×/week: 32.6 ± 39.4 μmol/L; Table 3 and Fig. 1). At study baseline we found 4 of these patients (31%) to be vitamin C deficient as compared to 6 (35%) in the intervention group. After 4 months into the study the fraction of vitamin C-deficient subjects decreased to 23 and 24%, respectively, in the control and treatment arms. At 12 months the fraction in the control arm decreased to 8% as compared to 35% in the intervention arm (Table 4). However, differences between the groups and within the groups over time were not significant.

**Table 1:** Demographics and clinical parameters of included subjects

Baseline characteristics of Daily Trial participants with vitamin C measures						
	All (*N* = 44)		Conventional Hemodialysis (*N* = 20)		Frequent Hemodialysis (*N* = 24)	
Characteristic	N Missing	^mean +/-SD^	N Missing	^a^	N Missing	^a^
Age (yrs.)	0	49.6 ± 12.1	0	50.6 ± 11.9	0	48.8 ± 12.4
Female sex (%)	0	16 (36%)	0	7 (35%)	0	9 (38%)
Hispanic ethnicity (%)	2	12 (29%)	2	3 (17%)	0	9 (38%)
Race	0		0		0	
Black White Native American		28 (64%)		14 (70%)		.14 (58%)
Aboriginal Canadian		4 (9%)		0		4 (17%)
Alaskan Native		3 (7%)		2 (10%)		1 (4%)
First Nation Asian Native Hawaiian		2 (5%)		1 (5%)		1 (4%)
Other Pacific Islander		1 (2%)		1 (5%)		0
Other/mixed/unknown		6 (14%)		2 (10%)		4 (17%)
Duration of end-stage renal disease (%) < 2 yr. 2–5 yr. > 5 yr	11	.12 (36%) 15 (45%) 6 (18%)	4	.7 (44%) 9 (56%) 0	7	.5 (29%) 6 (35%) 6 (35%)
Diabetes (%)	0	13 (30%)	0	6 (30%)	0	7 (29%)
Residual kidney function (%) Anuria > 0 to 1 ml/min > 1 to 3 ml/min	0	.26 (59%) 9 (20%) 9 (20%)	0	.6 (30%) 5 (25%) 9 (45%)	0	.20 (83%) 4 (17%) 0
Equilibrated per session Kt/V urea	0	1.44 ± 0.20	0	1.46 ± 0.22	0	1.43 ± 0.19
Predialysis albumin (g/dL)	0	4.12 ± 0.34	0	4.15 ± 0.36	0	4.09 ± 0.32
Predialysis sodium (mEq/L)	0	139 ± 3	0	140 ± 3	0	139 ± 3
Predialysis potassium (mEq/L)	0	4.92 ± 0.59	0	4.83 ± 0.47	0	4.99 ± 0.68
Predialysis calcium (mg/dL)	0	9.35 ± 0.74	0	9.51 ± 0.83	0	9.22 ± 0.65
Predialysis hemoglobin (mg/dL)	0	12.1 ± 1.3	0	12.4 ± 1	0	11.8 ± 1.4
Predialysis Parathyroid hormone (pg/mL)	0	408 [283,927]	0	389 [246,1039]	0	471 [289,927]
Physical Health Composite score	0	40.3 ± 11.0	0	39.7 ± 9.6	0	40.9 ± 12.2
Mental Health Composite score	0	47.6 ± 12.4	0	45.8 ± 10.1	0	49.1 ± 14.0
Iron administration (Y/N)	0	1 (2%)	0	1 (5%)	0	0
Erythropoiesis-Stimulating Agents (EPO equivalent units)	0	9250 [3138,22,875]	0	8750 [2913,27,625]	0	9250 [3138,19,750]
Left Ventricular Mass (g)	0	166 ± 65	0	179 ± 66	0	155 ± 63
CRP (mg/dL)	10	0.362 [0.221,0.964]	4	0.412 [0.242,0.986]	6	0.362 [0.221,0.932]

^a^Plus-minus values are means ±SD. Bracketed values are medians with interquartile ranges (chosen for skewness of data as per visual inspection of distribution)

**Table 2:** Vitamin C supplements over the course of the study in both treatment groups

Period	Supplemental Vitamin C Intake	3×/week Treatment Group			6×/week Treatment Group		
		Total # Patients with Records For Period (Total # Records For Period)	# Patients with Records Matching Intake Criteria During Period (%)	# Records Matching Intake Criteria (%)	Total # Patients with Records For Period (Total # Records For Period)	# Patients with Records Matching Intake Criteria During Period (%)	# Records Matching Intake Criteria (%)
Randomization to Month 4	none	20 (24)	14 (70.0%)	14 (58.3%)	24 (26)	18 (75.0%)	18 (69.2%)
	>0 to 100 mg		6 (30.0%)	10 (41.7%)		6 (25.0%)	8 (30.8%)
Month 4 to Month 8	none	18 (18)	13 (72.2%)	13 (72.2%)	22 (22)	16 (72.7%)	16 (72.7%)
	>0 to 100 mg		5 (27.8%)	5 (27.8%)		6 (27.3%)	6 (27.3%)
Month 8 to Month 12	none	15 (15)	12 (80.0%)	12 (80.0%)	21 (21)	18 (85.7%)	18 (85.7%)
	>0 to 100 mg		3 (20.0%)	3 (20.0%)		3 (14.3%)	3 (14.3%)

**Table 3:** Treatment effect of more frequent hemodialysis on left ventricular mass (LVM), the Physical Health Component Score as per SF-36 and the circulating plasma vitamin C levels in the study population

Variable	Trial	Trt	Observeld Data (Mean ± SD)			Adjusted Means and Treatment Effects (± SE or with 95% Confidence Intervals) *P*-value			
			Baseline	F4	F12	Month 4		Month 12	
.	.	.	.	.	.	Change from Baseline	Treatment Comparison (6× vs. 3×)	Change from Baseline	Treatment Comparison (6× vs. 3×)
LVM (g)	Daily	3×	179 ± 66	.	162 ± 75	–	–	−13.8 ± 9.3	−14.6 (−37.9, 8.7) *P* = 0.21
		6×	155 ± 63	.	135 ± 40	–		−28.4 ± 8.2	
PHC (SF36)	Daily	3×	38.9 ± 9.9	37.3 ± 9.1	38.2 ± 10.3	−.2.4 ± 1.5	+ 4.3* (0.5, 8.1) *P* = 0.027	−1.4 ± 1.8	+ 4.0 (− 0.7, 8.6) *P* = 0.093
		6×	41.8 ± 12.0	42.8 ± 11.1	43.6 ± 9.5	+1.9 ± 1.3		+ 2.6 ± 1.7	
Plasma Vitamin C Concentration (μmol/L)	Daily	3×	32.6 ± 39.4	49.4 ± 69.2	25.8 ± 21.7	20.8 ± 12.1	−26.2 (−57.5, 5.1) *P* = 0.10	−4.8 ± 6.2	−2.5 (− 15.6, 10.6) *P* = 0.70
		6×	25.8 ± 25.9	22.6 ± 17.5	22.4 ± 19.1	5.4 ± 11.0		−7.3 ± 5.7

**Table 4:** Prevalence of vitamin C deficiency in the study population at different timepoints during the study period

Treatment Group	Baseline	Month 4	Month 12
3× per week	4 / 13 (31%)	3 / 13 (23%)	1 / 13 (8%)
6× per week	6 / 17 (35%)	4 / 17 (24%)	6 / 17 (35%)

### Comparative analysis

In line with the analysis of prevalence of vitamin C deficiency and its evolution over the course of the study we also did not find significant adjusted treatment effects on vitamin C concentrations [− 26.2 (95%CI -57.5 to 5.1) μmol/L at Month 4 and − 2.5 (95%CI -15.6 to 10.6) μmol/L at Month 12 (Table 3). While consistent with the results of the main trial in terms of reductions over time, the models constructed for Physical Health Component score and left ventricular mass did not show significant treatment effects of more frequent HD for those participating in this ancillary study, likely due to the low sample size (Table 3).

### Correlational analysis

**Fig. 2:**
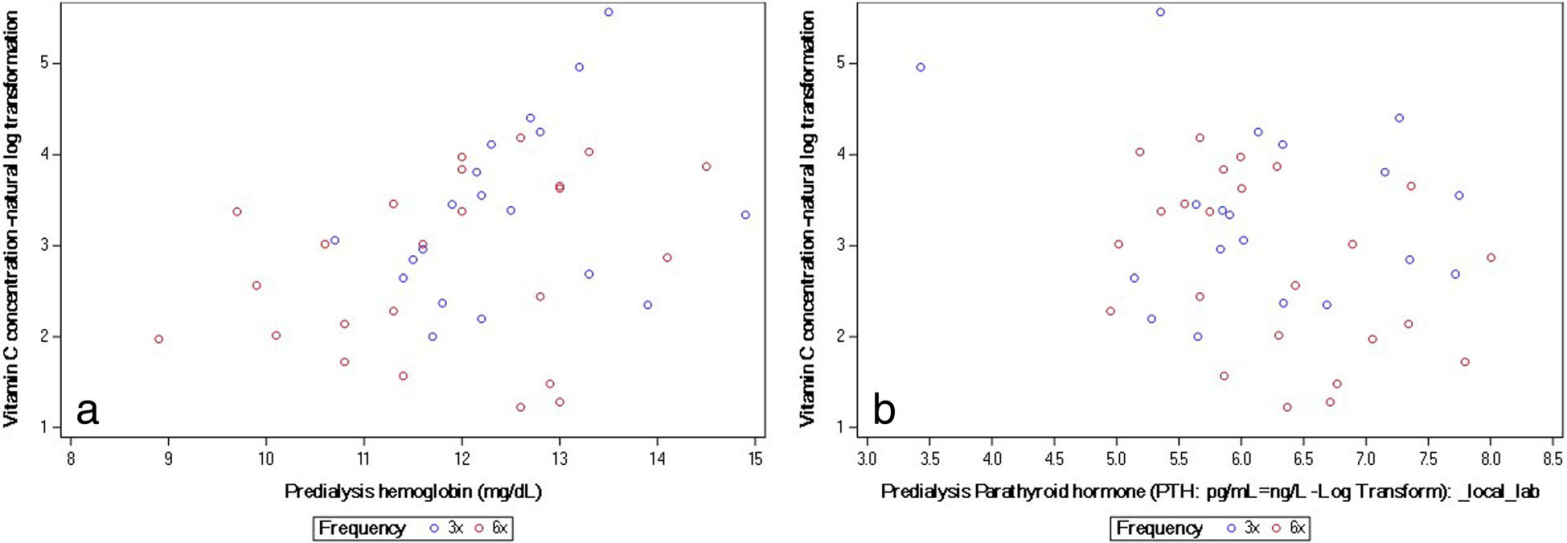
Relationship between (natural log transformed) plasma vitamin C and pre-dialysis **a**) hemoglobin and **b**) (log transformed) parathyroid hormone

In Pearson correlation predialysis vitamin C at baseline correlated significantly with those of predialysis hemoglobin (positive correlation; Spearman r = 0.3; *P* = 0.03) and PTH (negative correlation; Pearson r = − 0.3, *P* = 0.04), respectively (Table 5; Fig. 2a and Fig. 2b). No associations were found between vitamin C concentrations and CRP, Physical Health Component score and equilibrated non-normalized protein catabolic rate. In line with the primary hypothesis tested, we also found no associations between predialysis vitamin C concentrations and equilibrated per session Kt/V and weekly standard Kt/V (Table 5).

**Table 5:** Correlation of various parameters with hypothesized associations with circulating vitamin C levels

Correlations with Vitamin C Concentrations (Natural Log Transformed)							
Statistic / Grouping	CRP (mg/L) -natural log transformation	Predialysis hemoglobin (mg/dL)	Equilibrated per session Kt/V urea	ePCR (g/kg/d)	Predialysis Parathyroid hormone	Physical Health Composite score	Dialysis weekly standard Kt/V
Pearson correlation (complete cohort)	−0.10 (*P* = 0.59) [*N* = 34]	0.32 (*P* = 0.03) [*N* = 44]	− 0.05 (*P* = 0.77) [*N* = 44]	0.19 (*P* = 0.21) [*N* = 44]	− 0.31 (*P* = 0.04) [*N* = 44]	0.12 (*P* = 0.44) [*N* = 44]	− 0.07 (*P* = 0.64) [*N* = 44]
3×, Pearson correlation	−0.15 (*P* = 0.58) [*N* = 16]	0.35 (*P* = 0.13) [*N* = 20]	−0.07 (*P* = 0.78) [*N* = 20]	0.21 (*P* = 0.37) [*N* = 20]	−0.31 (*P* = 0.19) [*N* = 20]	0.35 (*P* = 0.13) [*N* = 20]	− 0.16 (*P* = 0.49) [*N* = 20]
6×, Pearson correlation	−0.08 (*P* = 0.76) [*N* = 18]	0.38 (*P* = 0.07) [*N* = 24]	−0.10 (*P* = 0.66) [*N* = 24]	0.12 (*P* = 0.58) [*N* = 24]	−0.36 (*P* = 0.09) [*N* = 24]	−0.21 (*P* = 0.32) [*N* = 24]	+ 0.07 (*P* = 0.76) [*N* = 24]
Spearman correlation (complete cohort)	−0.19 (*P* = 0.28) [*N* = 34]	0.34 (*P* = 0.02) [*N* = 44]	−0.08 (*P* = 0.63) [*N* = 44]	0.19 (*P* = 0.23) [*N* = 44]	−0.16 (*P* = 0.30) [*N* = 44]	0.10 (*P* = 0.52) [*N* = 44]	− 0.12 (*P* = 0.43) [*N* = 44]
3×, Spearman correlation	−0.31 (*P* = 0.25) [*N* = 16]	0.33 (*P* = 0.16) [*N* = 20]	−0.16 (*P* = 0.50) [*N* = 20]	0.09 (*P* = 0.70) [*N* = 20]	0.11 (*P* = 0.63) [*N* = 20]	0.47 (*P* = 0.04) [*N* = 20]	− 0.31 (*P* = 0.19) [*N* = 20]
6×, Spearman correlation	−0.07 (*P* = 0.77) [*N* = 18]	0.30 (*P* = 0.16) [*N* = 24]	−0.10 (*P* = 0.66) [*N* = 24]	0.12 (*P* = 0.58) [*N* = 24]	−0.40 (*P* = 0.06) [*N* = 24]	−0.22 (*P* = 0.30) [*N* = 24]	+ 0.04 (*P* = 0.86) [*N* = 24]

## Discussion

### Statement of principal findings

Based on data from our ancillary study from this large randomized-controlled trial, no significant treatment effect of the intervention on circulating plasma vitamin C concentrations and the prevalence of vitamin C deficient patients was found, allaying the concerns that more frequent hemodialysis would decrease the concentrations of water-soluble vitamins and adversely affect patients’ well-being. Of great interest for future studies, however, are the reported correlations between vitamin C and hemoglobin and PTH, respectively, that support the importance of vitamin C for normal bone and mineral metabolism and anemia management.

### Discussion in the light of other studies

#### Treatment effect of more frequent hemodialysis and dialysis dose

Morena and colleagues estimated the vitamin C diffusive flux at 271 μg/min and 66 mg average loss per 4-h session which can be a substantial amount of loss with increased dialysis frequency [18]. Boehm and colleagues investigated the vitamin C status in hemodialysis patients and reported a 50% decrease in vitamin C associated with dialysis treatment. Despite substantially higher weekly delivered dialysis dose (weekly standard Kt/V) in the more frequent hemodialysis arm (3.6 ± 0.6 versus 2.6 ± 0.3) [13] in our data, there was no effect on circulating predialysis vitamin C levels (Table 3 and Fig. 1) and the prevalence of vitamin C deficiency (Table 3). This can be explained by adequate nutritional intake throughout the study and the fact that most vitamin C is stored within the tissues which serve as a pool that refills the intravascular compartment from which dialysis removes compounds [19, 20].

#### Associations between vitamin C and hemoglobin

Vitamin C plays a major role in iron metabolism facilitating storage and iron mobilization thus affecting the levels of biologically available iron needed for hematopoiesis [21]. Studies in dialysis patients have shown that vitamin C supplementation may lead to decreased serum ferritin levels [22], which is consistent with storage iron mobilization. These reports are supported by previously reported data from several studies [22–28] and confirmed in a recently published meta-analysis by Deved and colleagues [29]. Our data on the correlation between hemoglobin and vitamin C supports this association and the claims made by Deved and others on the importance of considering the prescription of vitamin C in anemia management (Fig. 2a and Table 5).

#### Associations between vitamin C and PTH

An inverse relationship between vitamin C and PTH is in line with data reported by Richter and colleagues [17], which showed an inverse relationship between vitamin C and PTH concentrations. The authors hypothesized an effect of vitamin C on post-receptor events in the super-family of seven membrane spanning receptors [30], that include PTH receptors in the bone [31] and calcium-sensing receptors in the parathyroid gland [32]. Consistent with Richter et al.’s findings we also see an inverse relationship between the concentrations of these two compounds in our data (Fig. 2b and Table 5). The absence of alkaline phosphatase measurements in the data of the main trial did not allow the reproduction of that aspect of the study by Richter et al. [17].

#### Associations between inflammatory markers

Given the role of vitamin C as an antioxidant and scavenger of reactive oxygen species, we hypothesized a relationship between levels of vitamin C at baseline and of CRP that were collected as part of the protocol in the main trial, as was reported by another investigation [23]. In our data there was no clear relationship between CRP and vitamin C, which, however, does not necessarily imply a lack of effect of vitamin C on oxidative stress and the (increased) level of inflammation in dialysis patients.

### Strengths and limitations

While the sample size of our ancillary study is admittedly small, we have had the great advantage to follow recruitment and randomization on a facility basis of the main trial. Thus we claim that also our data can be considered quasi-randomized, minimizing confounding. However, recruitment only took place in the East Coast consortium, thus a possible lack of generalizability needs to be acknowledged that may limit the interpretation of our findings in different dialysis populations and for different practice patterns. A clear limitation of our analysis is the lack of dietary data and the lack of data in the West Coast consortium concerning supplements ingested by the studied patients. A good argument can, however, be made that diet and supplement intake have likely not changed during the study period and practices were balanced between the randomized groups.

## Conclusion

We found a lack of treatment effect of more frequent hemodialysis treatments on circulating plasma vitamin C concentrations and the prevalence of vitamin C deficiency. This is of importance for the nephrologist when considering increased dialysis frequency, additional dialysis treatments or extended dialysis session lengths for patients. The associations between vitamin C, and hemoglobin and PTH, respectively, may be relevant when prescribing or recommending vitamin C to dialysis patients, particularly to those receiving higher dialysis frequency or additional treatments, and/or are being prescribed with higher dialysis doses per dialysis session. The effect on both parameters requires consideration of the overall recommendations on vitamin C in dialysis patients, given that current concerns on conversion of ascorbate into oxalate and subsequent deposit in various tissues (including vessel walls leading to accelerated arterial stiffening) are based on outdated and small case series and reports [33–38]. Large controlled trials are needed to determine the optimal approach for vitamin C supplementation in dialysis patients. It would be prudent to ensure that patients who received extended or more frequent dialysis be prescribed a standard regimen containing 60 or 100 mg of vitamin C per day.

## Appendix

### FHN Trial Group

Chair, Steering Committee: Kliger A; NIDDK: Eggers P, Briggs J, Hostetter T, Narva A, Star R; Centers for Medicare and Medical Services: Augustine B, Mohr P; Data Coordinating Center – Cleveland Clinic: Beck G (PI), Fu Z, Gassman J, Greene T, Daugirdas J, Hunsicker L, Larive B, Li M, MacKrell J, Wiggins K, Sherer S, Weiss B; MRI Core – Ohio State University and Mt. Sinai Medical Center: Rajagopalan S, Sanz J, Dellagrottaglie S, Kariisa M; Tran T, West J; Central Quality of Life Core – University of Pittsburgh: Unruh M; Keene R, Schlarb J; Central Holter Core– Toronto General Hospital: Chan C; McGrath-Chong M; Biospecimen Repository – Fisher BioServices: Frome R, Higgins H, Ke S, Mandaci O, Owens C, Snell C; Data Safety and Monitoring Board: Eknoyan G (Chair), Appel L, Cheung A, Derse A, Kramer C, Geller N, Grimm R, Henderson L, Prichard S, Roecker E;

**Daily Trial Clinical Sites** – University of California San Francisco (UCSF)/Stanford Consortium: Chertow G (PI); UCSF and San Francisco Bay Area: James S, Chertow G, Tamura M, Hall Y, McCulloch C, Painter P, Gorodetskaya I, Tichy M, Humphreys M, Luan J, Escalada R, Rodriquez R; UC Davis and Sacramento Area: Depner T, Kaysen G, Suter M, Sonico J, Anderson S; El Camino Hospital and Satellite Health Care: Ting G, Schiller B, Coplon N, Doss S, Rogers J, Dominguez A, Atwal J, Lemus D; UCLA and Los Angeles Area: Rastogi A, Nissenson A, Goodman W, Salusky I, Schweitzer S, Rivas M, Smith M, Gayda P, Hernandez A, Rashid M; UCSD and San Diego Area: Mehta R, Pepas J, Bharti B, Nabali A, Manaster R, Mathew R, Shah S, Sanz G, Wei J; University of Texas, San Antonio: Ayus J, Achinger S, Gutierrez M; Renal Research Institute (RRI) New York Consortium: Levin N (PI); Bay W, Carter M, Geronemus R, Kuhlmann M, Handelman G, Gotch F, Finkelstein F, Kimmel P, Lacson E, Ornt D, Greenwood R, Vassalotti J, Burrowes J; RRI New York City: Levin N, Kotanko P, Kaufman A, Winchester J, Meisels I, Radbill B, Chang J, Fofie Y, Ramos R, Sergeyeva O, Callegari J, Arthur B, Tarallo M, Ulloa D, Apruzzese R; University of Western Ontario: Lindsay R, Suri R, Garg A, Bullas R Mazzorato A; Wake Forest University School of Medicine: Rocco M, Burkart J, Moossavi S, Mauck V, Kaufman T, Coppley A; Vanderbilt University Medical Center: Schulman G, McLeroy S, Sika M, Leavell E; Barnes Jewish/Washington University: Miller B, Schussler R, Bardsley J, Skelton R.

**Nocturnal Trial Clinical Sites** – Wake Forest University School of Medicine Consortium: Rocco M (PI); Barnes-Jewish/Washington University: Miller B, Riley J, Schuessler R; Lynchburg Nephrology: Lockridge R, Pipkin M, Peterson C; Rubin Dialysis: Hoy C, Fensterer A, Steigerwald D; University of Iowa: Stokes J, Somers D, Hilkin A, Lilli K, Wallace W, Franzwa B, Waterman E; University of Toronto: Chan C, McGrath-Chong M; University of British Columbia: Copland M, Levin A, Sioson L, Cabezon E, Kwan S, Roger D; University of Western Ontario: Lindsay R, Suri R, Champagne J, Bullas R, Garg A, Mazzorato A, Spanner E; Wake Forest University School of Medicine: Rocco M, Burkart J, Moossavi S, Mauck V, Kaufman T; Humber River Regional Hospital: Pierratos A, Chan W, Regozo K, Kwok S.

### Recruitment sites

**Renal Research Institute Consortium**: Irving Place Dialysis Center, Queens Artificial Kidney Center, South Queens Dialysis Center, Southern Manhattan Hemodialysis Center, St. Alban’s Dialysis, Upper Manhattan Dialysis Center, Yorkville Dialysis Center.

## Abbreviations

CRP: C-reactive protein
eKt/V: equilibrated Kt/V
ePCR: equilibrated Protein Catabolic Rate
FHN: Frequent Hemodialysis Network
HD: Hemodialysis
HPLC: High-performance liquid chromatography
NIDDK: National Institutes of Diabetes and Digestive and Kidney Diseases
NIH: National Institutes of Health
PTH: Parathyroid hormone
USA: United States of America
